# Rapid *in vitro* evolution of flucytosine resistance in *Candida auris*

**DOI:** 10.1128/msphere.00977-24

**Published:** 2025-03-18

**Authors:** Trinh Phan-Canh, Duc-Minh Nguyen-Le, Phuc-Loi Luu, Narakorn Khunweeraphong, Karl Kuchler

**Affiliations:** 1 Max Perutz Labs, Vienna Biocenter Campus (VBC) 249082 , Vienna, Austria; 2 Medical University of Vienna, Center for Medical Biochemistry 27271 , Vienna, Austria; 3 Institute for Applied Research in Health Sciences and Aging (ARiHA)-Thong Nhat Hospital 434429 , Ho Chi Minh City, Vietnam; 4 Mathematics Department, Faculty of Fundamental Sciences, University of Medicine and Pharmacy at Ho Chi Minh City, Ho Chi Minh City, Vietnam; University of Guelph, Guelph, Ontario, Canada

**Keywords:** *Candida auris*, flucytosine, 5FC, drug resistance, evolution, *FCY2*, *FUR1*

## Abstract

The pan-antifungal-resistant pathogen *Candida auris* has been causing high-mortality infection outbreaks in hospitals and healthcare settings. The prodrug 5-fluorocytosine (5FC) is one of four chemical entities, but its clinical use as an antifungal drug has been limited owing to pronounced resistance. However, antifungal combination therapy with 5FC appears as a promising strategy for treating *C. auris* infections. Here, we show that a *C. auris* clinical isolate can rapidly acquire genetic mutations to mount 5FC resistance after only one to two passages under drug selection. We exploit a new bioinformatics workflow to identify genetic polymorphisms from RNA-seq data. Strikingly, we identify several mutations in the *FUR1* gene encoding the 5-fluorouracil convertase that normally generates the active drug. A single nonsense mutation truncates the enzyme at residue Q30*, leading to 5FC resistance due to inactive Fur1. Whole-genome sequencing analysis revealed that an indel mutation in *FCY2* also contributes to 5FC resistance. Furthermore, at least one out of seven adapted strains acquired enhanced 5FC tolerance without mutations in the 5FC conversion pathway. Thus, we demonstrate that *FUR1* mutations are critical drivers of 5FC resistance in *C. auris*.

**IMPORTANCE:**

*Candida auris* is a high-priority human fungal pathogen, causing infection outbreaks of high mortality in healthcare settings. Antifungal combination therapy with 5-fluorocytosine (5FC) is one of the emerging approaches in treatment. However, acquired 5FC resistance traits have been a matter of concern. 5FC is taken up by fungal cells via a cytosine permease and further metabolized by a cytosine deaminase to 5-fluorouracil (5FU). 5FU is then converted by the Fur1 uracil phosphoribosyltransferase into a toxic antimetabolite that disrupts fungal RNA and DNA syntheses. Mutations in these proteins are commonly associated with 5FC resistance in fungal species. Here, we show that *C. auris* can rapidly develop resistance under 5FC selective stress owing to mutational inactivation of Fur1 function. Moreover, other mechanisms that bypass mutations in the 5FC conversion pathway may also contribute to 5FC resistance traits. Finally, we have developed a tailored bioinformatics workflow that facilitates the identification of polymorphisms associated with 5FC resistance in clinical isolates.

## INTRODUCTION

The human fungal pathogen *Candida auris* has been causing serious infection outbreaks worldwide (1, 2). Hence, the Centers for Disease Control and Prevention (CDC) and WHO declared it as a critical fungal pathogen due to its multidrug resistance (MDR), as well as extreme adhesion to skin and abiotic surfaces (1–3). MDR poses one of the biggest challenges in antifungal therapy. Current clinical practice relies on echinocandins as first-line treatment for most *C. auris* infections. However, echinocandin resistance increases, especially during treatment (4–6), often resulting in therapeutic failure for recurrent infections (7, 8). Furthermore, the poor distribution of echinocandins into the urinary tract and central nervous system limits their use for treating related infections. Clinical resistance to azoles and amphotericin B (AMB) is alarmingly high, as they reach ~90% and up to 30%–50%, respectively (9). Most concerningly, some *C. auris* clinical isolates acquire resistance to all antifungal drug classes (10–13). Of note, combinatorial regimens *in vitro* such as 5-fluorocytosine (5FC) with AMB or echinocandin (14–16) promise better therapeutic efficiency. Indeed, a combination therapy for *C. auris* infections with 5FC has been an emerging treatment option in clinical settings (13, 14, 17).

5FC was approved by the US Food and Drug Administration for antifungal treatment in 1971 and was a recognized option for cryptococcal meningitis and systemic candidiasis during the 1970s. However, the rapid emergence of resistance to 5FC monotherapy has limited its clinical use (18, 19). Indeed, many *C. auris* clinical isolates, particularly those from clade I, exhibit pronounced resistance to 5FC (13, 14, 20). 5FC is a prodrug requiring cellular uptake mediated by the cytosine permease Fcy2. Inside cells, the fungal-specific cytosine deaminase Fcy1 (21) converts the prodrug into the active antimetabolite 5-fluorouracil (5FU). The Fur1 phosphoribosyltransferase (UPRTase) converts 5FU into a toxic metabolite that disrupts RNA and DNA syntheses (21). 5FC resistance mechanisms are well-characterized in other fungal pathogens, primarily involving loss-of-function mutations in the *FCY2-FCY1-FUR1* pathway (18, 21–23). In *C. auris*, some clinical isolates harbor specific mutations associated with 5FC resistance, including *ADE17*:G45V + *FUR1*:1∆33 and CrcB:V119L + *FCY2*:M128fs (13). Additionally, the mutant variant, *FUR1:*F211I, emerged as a 5FC-resistant clinical isolate in a UK outbreak (24).

The use of 5FC in combination therapies appears as a promising approach, particularly in cases of echinocandin resistance, recurrent infections, or infections of body sites with poor echinocandin distribution such as the central nervous system or the urinary tract. However, the precise molecular mechanisms underlying 5FC resistance in *C. auris* remain incompletely understood. In this study, we show that different degrees and levels of acquired 5FC resistance emerge in distinct clones after a short-term *in vitro* evolution under selective pressure. We demonstrate that several mutations in *FUR1* and *FCY2* can confer marked 5FC resistance.

## RESULTS

### Rapid *in vitro* evolution of 5FC resistance in *C. auris*

**Fig 1:**
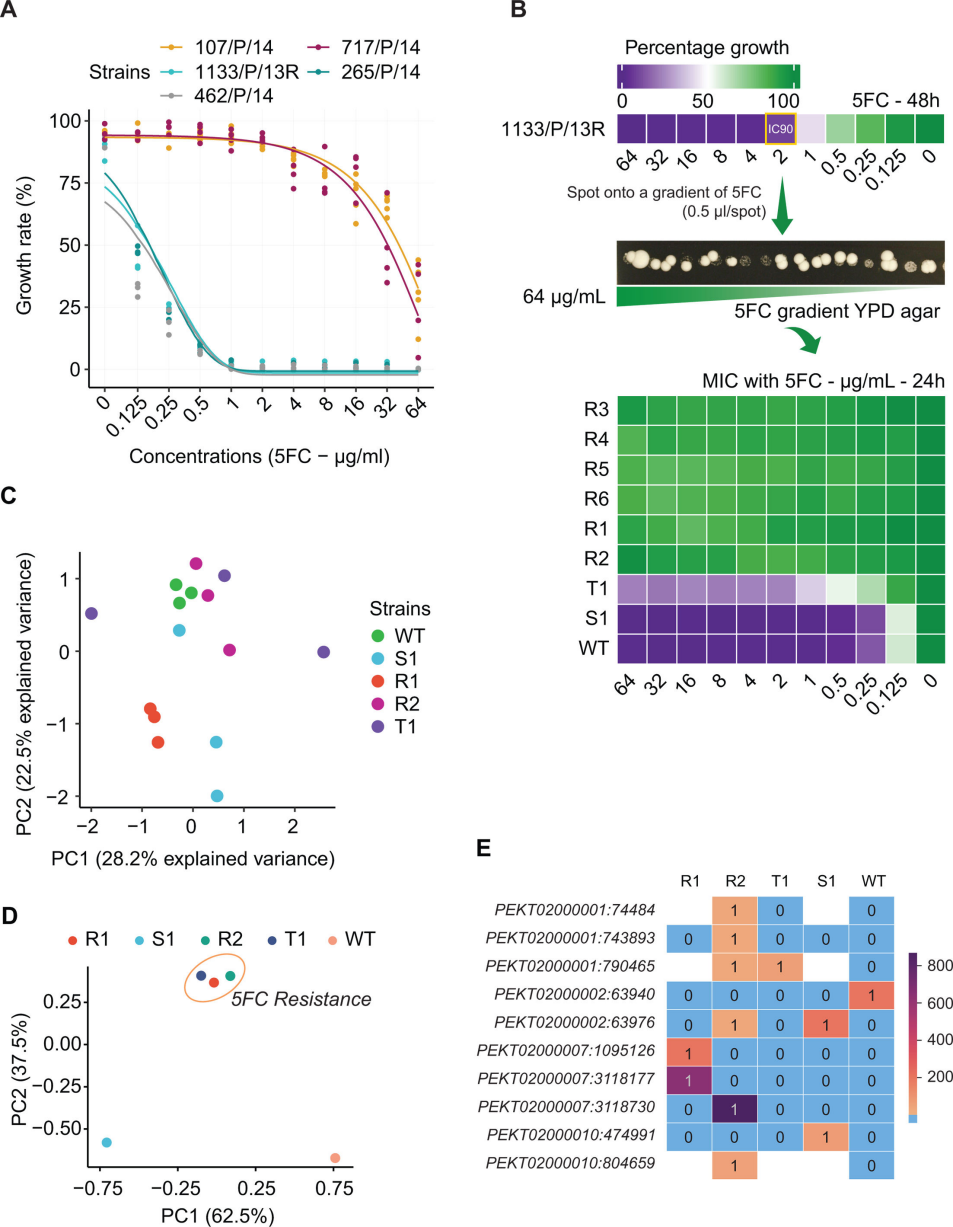
*In vitro* evolution of 5FC resistance in *C. auris*. (**A**) Dose response assays for 5FC with different *C. auris* clinical isolates. The lines represented predicted fitting curves using a four-parameter model from the *drc* R package. (**B**) Rapid evolution of 5FC resistance in MDR *C. auris* strains. (**C**) Principal component analysis (PCA) of 5FC-resistant and 5FC-sensitive clones based on gene expression data. 1133/P/13R–wild-type strain (WT); R1-R6: *in vitro* adapted 5FC-resistant clones; T1: clone showing 5FC tolerance; S1: 5FC-sensitive clone. (**D**) PCA for single nucleotide polymorphisms (SNPs) passing specific filtering criteria. (**E**) Comparative SNP profiling of *C. auris* strains against the susceptible wild type control (WT) strain; 1: refers to genotype-matching alternate allele compared to reference genome; 0: genotype is the same as the reference allele in the *C. auris* B8441 strain. Empty white spaces illustrate missing or unidentified genotypes in corresponding samples. The color bar on the right shows the number of alternate-supporting reads covered for a specific variant. At least three biological replicates were performed for MIC quantification and RNA-seq experiments.

As previously reported, 3%–14% of clade I *C. auris* clinical isolates are resistant to 5FC (14, 20) based on the Clinical & Laboratory Standards Institute (CLSI) breakpoint from other *Candida* species (25). Of note, 5FC-resistant strains were isolated from a patient post-5FC treatment (13, 26), suggesting a high propensity for rapid emergence of acquired 5FC resistance to this antifungal drug in *C. auris*. Therefore, we first conducted minimum inhibitory concentration (MIC) assays for 5FC with different clinical isolates from this clade, following the CLSI guidelines (27). Our results indicated that two strains, 107/P/14 and 717/P/14, exhibited significantly higher MICs when compared to others (1133/P/13R, 265/P/14, and 462/P/14) (Fig. 1A). To mimic conditions under which MDR strains can acquire pan-resistance traits, we conducted a short-term evolutionary assay using the 5FC-sensitive strain 1133/P/13R (wild type [WT]). This strain displays low susceptibility to AMB, echinocandin, as well as azoles (28, 29).

Briefly, the WT strain was cultured in different 5FC concentrations for 48 hours. The well at IC90 appearing after 48 hours was spotted onto yeast extract-peptone-dextrose (YPD) agar containing a gradient of 5FC from 0 to 64 µg/mL to select for resistant clones (Fig. 1B). Interestingly, some adapted 5FC-resistant clones with remarkably high fitness emerged after 4 days on selective plates. Eight random clones were re-isolated onto YPD agar for 2 days and preserved in 25% glycerol at −80°C for further experiments. These adapted *C. auris* clones were subjected to MIC assays with 5FC, with the WT strain serving as a control. Six clones were found to have acquired high 5FC resistance with MICs >64 µg/mL (R1-6; R, resistant). Of note, clone T1 exhibited an MIC ~1 µg/mL but showed a remarkable tolerance to even higher concentrations (T, tolerance) (Fig. 1B). These resistant clones emerged after only one to two passages in medium containing 5FC, indicating a capacity for rapid acquisition of 5FC resistance in *C. auris* (13, 26). This may explain the evolution of pan-antifungal resistance affecting all four clinical antifungal classes as observed in the transplant patients (13, 16, 26).

### A modified bioinformatic workflow for SNP calling from RNA-seq data

Because the resistant clones emerged after only one to two passages, and since the MIC levels varied across clones, we first hypothesized that this effect might be associated with mechanisms linked to epigenetic adaptation and transcriptional regulation. Thus, we randomly selected two resistant (R), one tolerant (T), and one sensitive (S) clone for RNA-seq analysis using the same workflow as previously described (28, 29). Unexpectedly, principal component analysis (PCA) revealed that samples from different phenotypes (R, T, and S) were not clearly separated (Fig. 1C). Differential gene expression analysis between the different resistant and sensitive phenotypes showed no consistent pattern in differentially expressed genes (Fig. S1). Therefore, it appears unlikely that transcriptional dysregulation is the major contributor to 5FC resistance. Alternatively, 5FC exposure in *C. auris* may drive polymorphic genetic variations manifesting 5FC resistance.

**Fig 2:**
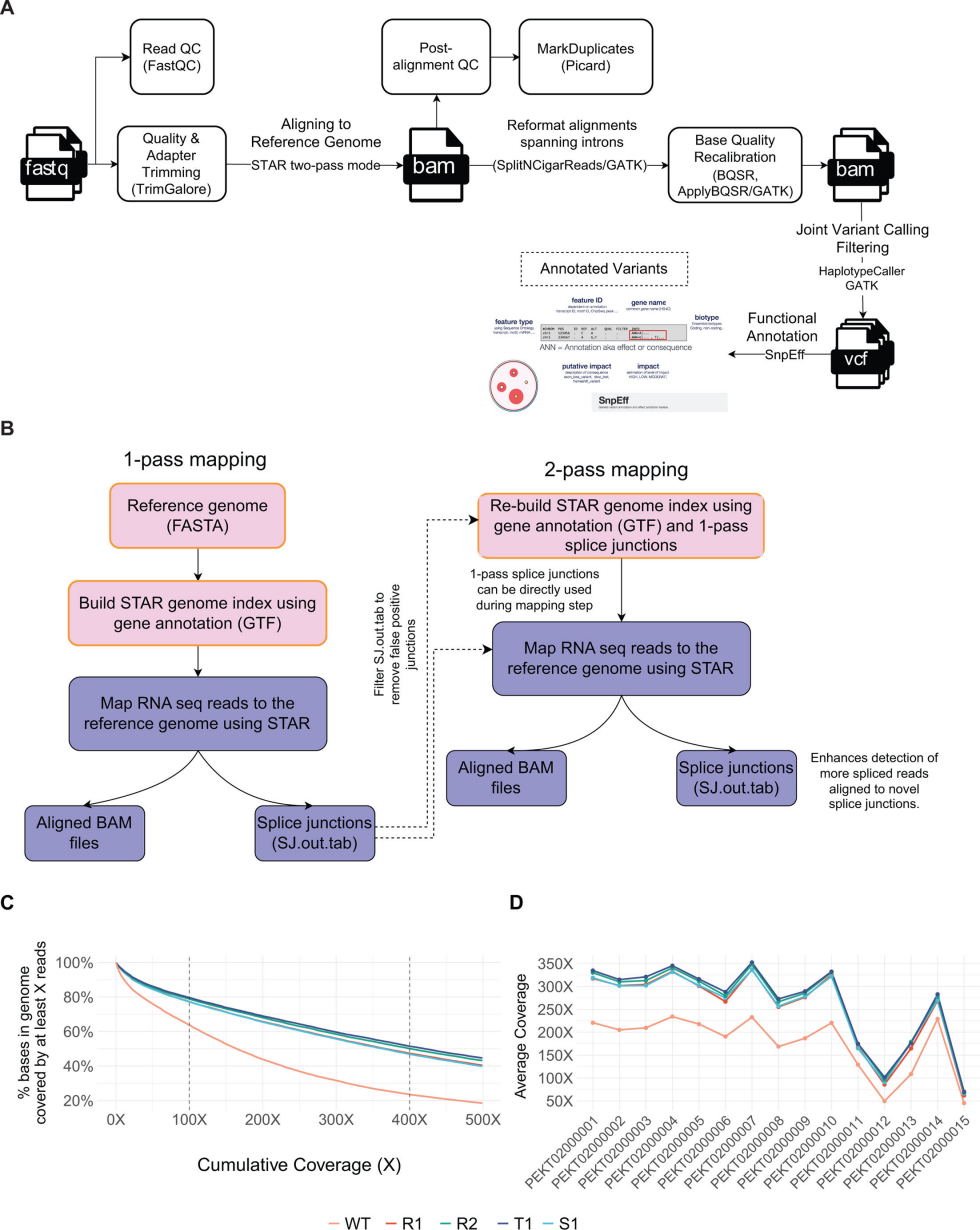
Computational workflow for SNP variant calling from RNA-seq data. (**A**) Bioinformatics workflow for SNP calling from RNA-seq data. (**B**) A two-pass STAR alignment workflow increases the number of spliced mRNA reads against novel splice junctions. (**C**) Cumulative coverage distribution across all samples. (**D**) Fluctuating coverage per scaffold.

Since RNA-seq analysis failed to uncover potential causative mechanisms of 5FC resistance, we developed a new bioinformatics workflow to identify potential SNPs from RNA-seq data (Fig. 2A). Of note, we applied the STAR two-pass mode (30) for aligning to a reference genome to enhance alignment accuracy around newly discovered splice junctions (Fig. 2B). We achieved an average coverage depth of 150×–200× for each scaffold, except for PEKT02000012 and PEKT02000015, which had a coverage of 50×–100× (Fig. 2C and D). This coverage depth is considered high quality and sufficient for reliable SNP calling (Table S1 and S2). To minimize redundancy and potential confounding effects, we applied a pruning strategy for SNPs (31), maintaining approximate linkage equilibrium to prevent strong impacts of SNP clusters (32). The PCA analysis on the cleaned data showed that resistant clones clustered very well and separated from the sensitive strains WT and S1 (Fig. 1D). Importantly, we identified numerous potential SNPs in the resistant clones (Table 1), with the 5FC-resistant clone R2 harboring a high number of mutations (Fig. 1E).

**TABLE 1:** Potential genetic variants corresponding to peptide changes in adapted clones compared to WT

Strain	Ref	Scaffold:Pos	GeneID	Gene	Effect	cDNA	aa	Method
R1	WT	PEKT02000007:1095126	B9J08_003129		Downstream	160G^*a*^ > T		RNA-seq
R1	WT	PEKT02000007:3118177	B9J08_004076	*FUR1*	Missense	641G > C	R214T	RNA-seq, Sanger
R2	WT	PEKT02000002:63976	B9J08_000526		Missense	1795A > G	T599A	RNA-seq
R2	WT	PEKT02000010:804659	B9J08_005267	*RBF1*	Missense	81A > T	Q27H	RNA-seq
R2	WT	PEKT02000007:3118730	B9J08_004076	*FUR1*	Truncation	88C > T	Q30^*a*^	RNA-seq, Sanger
R2	WT	PEKT02000001:74484	B9J08_000030	*RNT1*	Promoter	−439T > A		RNA-seq
R2	WT	PEKT02000001:743893	B9J08_000367	*ERG25*	Promoter	−483C > T		RNA-seq
R2^*a*^	WT	PEKT02000001:790465	B9J08_000387; B9J08_000388	tRNA; *NDT80*	Intergenic	−1368T > A		RNA-seq
T1^*a*^	WT	PEKT02000001:790465	B9J08_000387; B9J08_000388	tRNA; *NDT80*	Intergenic	−1368T > A		RNA-seq
S1	WT	PEKT02000010:474991	B9J08_005113	*TFB2*	Promoter	−387A > T		RNA-seq
S1	WT	PEKT02000002:63976	B9J08_000526		Missense	1795A > G	T599A	RNA-seq
R6	WT	PEKT02000006:444342	B9J08_002435	*FCY2*	Missense	650T > G	I217S	WGS^*c*^
R6	WT	PEKT02000006:444356	B9J08_002435	*FCY2*	Missense	636T > G	I212M	WGS
R6^*b*^	WT	PEKT02000008:8518	B9J08_004105		Missense	482A > T	D161V	WGS
R6^*b*^	WT	PEKT02000008:8523	B9J08_004105		Missense	487A > G	N163D	WGS
T1	WT	PEKT02000008:8654	B9J08_004104–B9J08_004105		Intergenic	−2887A > T		WGS
T1	WT	PEKT02000008:8662	B9J08_004104–B9J08_004105		Intergenic	−2895T > C		WGS
T1	WT	PEKT02000004:842355	B9J08_001924	*SEC27*	Upstream	−2996A > G		WGS
R6	WT	PEKT02000006:444356	B9J08_002435	*FCY2*	Missense	636_663delins-5´-GGCCATACGGCAGATGATG GCAATGAAA-3´	I212_A221delinsMAIRQMMAMK	Sanger
R3	WT	PEKT02000007:3118730	B9J08_004076	*FUR1*	Truncation	88C > T	Q30^*a*^	Sanger
R4	WT	PEKT02000007: 3118685	B9J08_004076	*FUR1*	Frameshift	Δ4nt175fs		Sanger
R5	WT	PEKT02000007:3118730	B9J08_004076	*FUR1*	Truncation	88C > T	Q30^*a*^	Sanger

^*a*^ This SNP was identified only from the RNA-seq data.

^*b*^ These variants were not present in the genome of R6, as confirmed by Sanger sequencing.

^*c*^ WGS, whole-genome sequencing.

### Mutations in *FUR1* confer 5FC resistance

**Fig 3:**
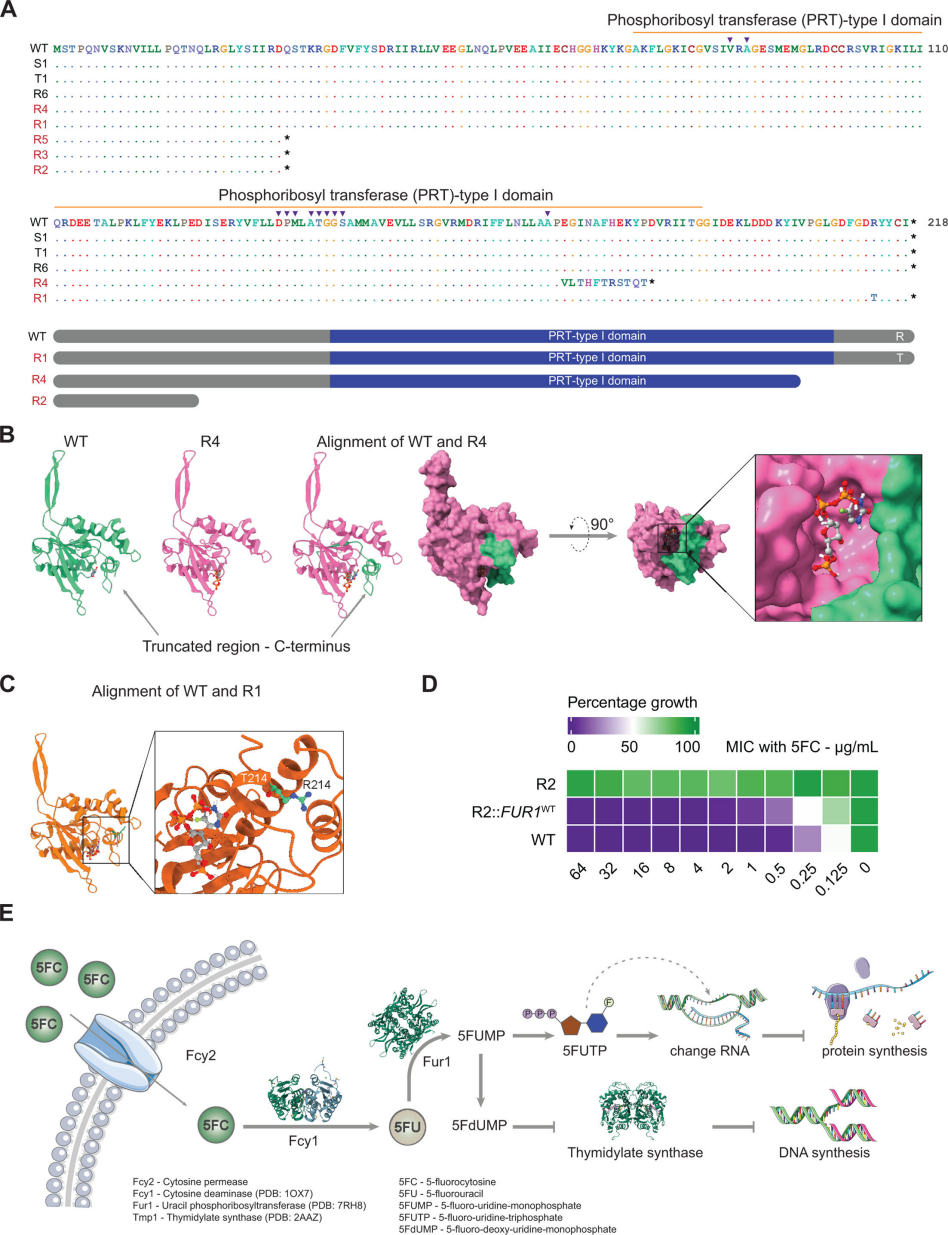
Lack of Fur1 function causes 5FC resistance in *C. auris*. (**A**) Fur1 amino acid sequences translated from Sanger sequencing data of *FUR1* of different *C. auris* clones (top). The schemes show different Fur1 peptides that may be obtained from mutated clones (bottom). ▼ indicates active site residues; * indicates a stop codon. (**B**) The homology models of Fur1 from WT and R4 show that the variant Δ4nt175fs does not directly affect the binding pocket for 5FU and PRPP. (**C**) Alignment of Fur1 from WT and R1 shows that the R214T mutation does not directly affect the 5FU-binding site. (**D**) MIC assays for resensitized clones show that the variant Q30* is responsible for Fur1-dependent 5FC resistance. Data from three biological replicates. (**E**) Pathway for 5FC uptake and conversion in fungi. Panel **E** uses modified drawings sourced from https://bioicons.com/.

Most interestingly, 5FC-resistant clones harbored several protein-altering mutations in the *FUR1* gene (Table 1) encoding a UPRTase that uses 5FU to produce 5FUMP (Fig. 3E) (18, 21, 33, 34). Notably, both resistant clones (R1 and R2) possess mutations in *FUR1*. Clone R1 has a missense mutation R214T, while clone R2 has the nonsense mutation Q30* in *FUR1* (Table 1). The variant Q30* may lead to an unstable *FUR1* transcript, as indicated by the downregulation of *FUR1* in clone R2 in comparison with WT or S1 (Fig. S1B).

To verify whether other mutations exist in this locus, we amplified the *FUR1* genes from the genomic DNA of all 5FC-adapted clones for Sanger sequencing (Fig. 3A). The variant Q30* was present in three out of eight tested clones (R2, R3, and R5), indicating that *in vitro* evolution favors a high frequency of this mutation in 5FC-resistant strains. Additionally, clone R4 acquired the mutation *FUR1*:∆4nt175fs, and clone R1 acquired R214T. Regarding the R214T mutation, the arginine to threonine change occurs at a highly conserved positively charged residue across eukaryotic and prokaryotic pathogens (Fig. S2A). These Fur1 residue changes in R1 and R4 are located at the C-terminal arm stretching from residues 175 to 218 near the binding pocket for 5FU (Fig. 3B and C). Both R4 and R1 are highly resistant to 5FC with MICs greater than 64 µg/mL. Indeed, the mutant variant F211I in a 5FC-resistant *C. auris* clinical strain (24) confirms a potential role of this domain in 5FC resistance. Furthermore, single nucleotide changes in *FUR1* are associated with 5FC resistance in *Candida albicans* and *Candida lusitaniae* (23, 35), supporting the link between resistance phenotypes of the R214T variant in strain R1, as well as the presence of a C-terminal-truncated Fur1 in R4.

Because the variant Q30* is of high frequency (three out of eight clones), we replaced this mutation in the R2 by *FUR1*^WT^. As expected, R2::*FUR1*^WT^ is resensitized to 5FC (Fig. 3D). Nonetheless, we observed that the R2::*FUR1*^WT^ is slightly tolerant to 5FC (Fig. 3D & 5A), implying as yet unknown mechanisms behind this phenomenon.

### A mutation in the *FCY2* homolog (B9J08_002435) may confer 5FC resistance

Although mutations in *FUR1* are major contributors, they are not the sole drivers of 5FC resistance. Notably, we did not detect *FUR1* mutations in strains R6 and T1, despite their elevated MICs (>64 µg/mL for R6) (Fig. 1B and 3A). To search for possible underlying mechanisms of 5FC resistance in strain R6, we subjected the strains R6, T1, as well as the parental WT strains to whole-genome sequencing (WGS).

**Fig 4:**
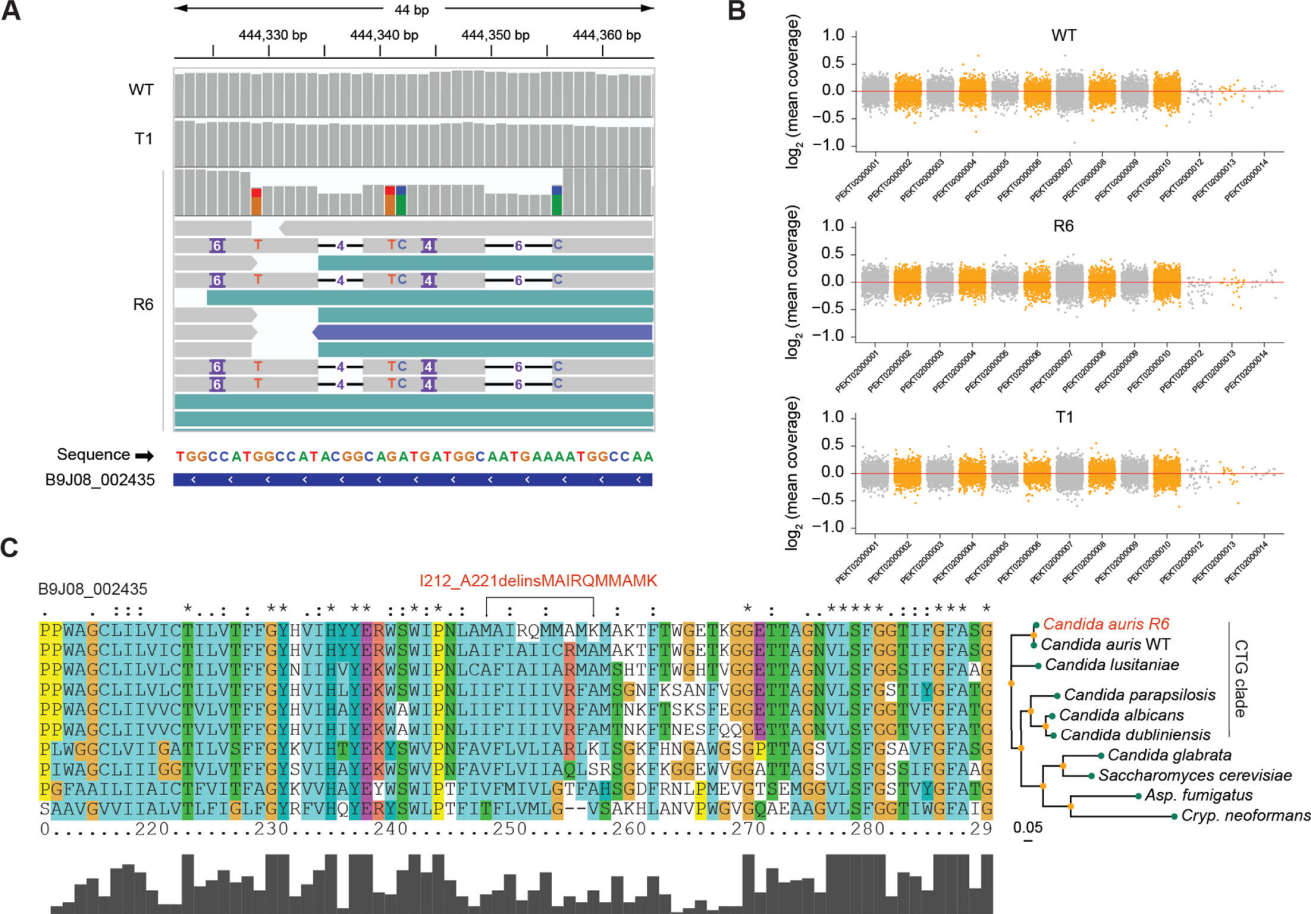
A mutation in the *FCY2.2* homolog (B9J08_002435) contributes to 5FC resistance.** (A**) IGV viewer reveals an indel mutation in the *FCY2.2* homolog of R6. (**B**) CNV analysis for WT, R6, and T1. CNVs are visualized as elevated coverage at the relevant loci. The *x*-axis represents the scaffold number, while the *y*-axis displays the log_2_ ratio (observed coverage/expected coverage). Each dot corresponds to an average bin size of 550 nucleotides. (**C**) Multiple sequence alignment and phylogenetic tree of Fcy2 sequences across fungal species. A snapshot around the mutational region was visualized.

The CDC MycoSNPs pipeline for the WGS workflow (29) failed to detect any variants with alternate allele fractions of 80% (ALT reads). However, reducing this threshold to >40% ALT reads revealed missense mutations in the loci B9J08_004105 and B9J08_002435, the latter being a homolog of *FCY2* (Table 1). *FCY2* encodes a cytidine transmembrane transporter that facilitates 5FC uptake into fungal cells (Fig. 3E), and mutations in *FCY2* confer 5FC resistance in other fungal species (33, 36–38). Visualization of *FCY2* using the Integrative Genomics Viewer (IGV) tool showed complex indel mutations from nucleotide positions 636–663, with approximately 50% ALT reads (Fig. 4A). However, no copy number variants (CNVs) were observed (Fig. 4B).

For validation, we amplified the regions of B9J08_004105 and *FCY2* from the WT and R6 strains and performed Sanger sequencing. This analysis confirmed the presence of the variant, I212_A221delinsMAIRQMMAMK in *FCY2*, which affects 10 amino acids conserved across all CTG clade *Candida* species (Fig. 4C). No mutations were detected in B9J08_004105 with Sanger sequencing (Table 1). Therefore, the resistance phenotype of R6 may primarily arise from this mutation in *FCY2*.

### 5FC tolerance is associated with altered gene expression profiles

**Fig 5:**
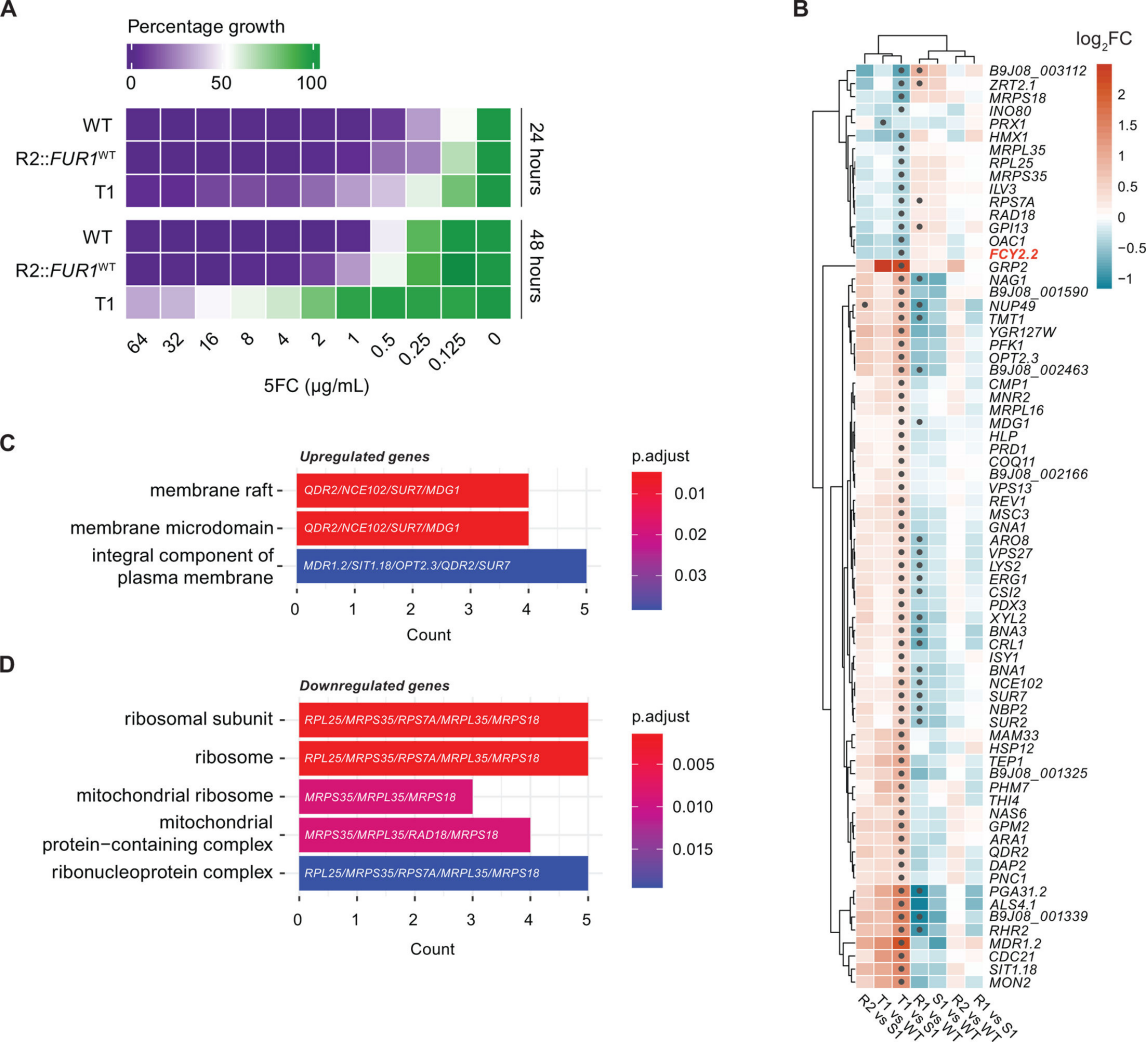
The 5FC tolerance is associated with gene expression profiles. (**A**) MIC assays for strains exhibiting 5FC tolerance. Three biological replicates were performed. (**B**) Heatmap with hierarchical clustering of comparisons between tolerant and sensitive strains. Circle dots indicate differentially expressed genes (adjusted *P*-value < 0.05). (**C and D**) Gene ontology (GO) enrichment analysis using the *enrichGO* function from the *clusterProfiler* R package. Upregulated (**C**) and downregulated (**D**) genes between T1 and S1 are shown. Cellular component terms are visualized.

As mentioned above, T1 appears to exhibit a higher tolerance (Fig. 5A), showing a remarkably increased MIC to 5FC at 48 hours when compared to 24 hours. A similar, albeit slight effect was observed in R2::*FUR1*^WT^, implying potential shared mechanisms overlapping between R2 and T1. Notably, we did not detect any SNP variants in coding sequences of T1 (Table 1), indicating that 5FC tolerance may be affected by gene dosage or altered protein levels rather than genetic variations.

Given a shared 5FC tolerance mechanism between R2 and T1, we integrated RNA-seq data from R2 and T1 comparisons with data from sensitive control strains (WT and S1). This analysis identified a cluster of upregulated genes in T1 (Fig. 5B and C), including membrane proteins and transporters such as *MDR1.2* (*B9J08_004113*), *SIT1.18* (*B9J08_004475*), *QDR2* (*B9J08_002663*), and *OPT2.3* (*B9J08_004544*). These genes participate in drug transport across cell membranes, suggesting that alterations in membrane functions may affect drug uptake and efflux to enable *C. auris* survival under 5FC stress. Of note, genes encoding ribosomal proteins were downregulated in T1 (Fig. 5D), implying changes in translational efficiency, which would explain why no striking effects were observed at the RNA expression level.

Interestingly, another as yet uncharacterized homolog of *FCY2* (B9J08_003017 – FCY2.2) was downregulated in T1 (Fig. 5B), providing an additional explanation for enhanced tolerance of T1 to 5FC. A similar trend was observed in R2, though the effect was minimal and not statistically significant, confirming the lower tolerance in R2::*FUR1*^WT^ when compared to T1 (Fig. 5A).

## DISCUSSION

While combination therapy with 5FC for treating *C. auris* infections is a promising therapeutic option (14–16), it emphasizes the need to identify and monitor potential resistance mechanisms. Here, we use an *in vitro* evolution approach to demonstrate that 5FC resistance can rapidly evolve after just one to two passages in a medium with 5FC selection. By integrating both RNA and DNA sequencing data, we uncover distinct mechanisms contributing to 5FC resistance in six adapted clones emerging after *in vitro* evolution. Our initial approach relied upon transcriptomic data, but differential gene expression analysis alone failed to yield comprehensive insights into mechanisms of 5FC resistance. To address this gap, we developed a new bioinformatics workflow enabling the identification of genetic variants directly from RNA-seq data sets. Our variant calling method is reliable and fully validated by the MycoSNPs pipeline from the CDC (39), which demonstrated solid consistency for the resulting mutant variants (Fig. S2B). The sensitive workflow also allows for identification of new variants associated with 5FC resistance in *FUR1* (Table 1).

Previous studies report mutations in *FUR1* in *C. auris* clinical strains, including *FUR1*:1∆33 and the F211I variant, both associated with 5FC resistance (13, 24, 26). In this study, the mutational analysis and genetic reversal experiments firmly establish that Fur1 is pivotal for 5FC resistance in *C. auris*. Specifically, we identify three novel *FUR1* variants (Q30*, R214T, and *FUR1*:∆4nt175fs) that are associated with 5FC resistance. Resistance phenotypes persist, even in the absence of missense or nonsense mutations in other loci. This suggests that even subtle changes in the *FUR1* sequence can influence drug susceptibility, highlighting a complex mechanism that may not only result from alterations in drug-binding affinities (35, 40).

Furthermore, whole-genome analysis of strain R6 revealed a novel indel mutation present in *FCY2* (B9J08_002435), resulting in a variant within 10 residues (I212_A221delinsMAIRQMMAMK) of Fcy2. Importantly, no other protein-altering mutations are detectable, strongly suggesting that this *FCY2* variant is a primary driver of 5FC resistance in strain R6. Indeed, two clinical strains harboring *CrcB*:V119L + *FCY2*:M128fs mutations have 5FC MIC values of ~256 µg/mL (13), which is consistent with our notion that *FCY2* is associated with 5FC resistance in *C. auris* in addition to *FUR1* mutations.

The integrated data also suggest tolerance traits in strain T1 and R2::*FUR1*^WT^. While the current data do not allow us to determine the precise mechanisms causing this phenomenon, our sequencing data lack a support for genetic mutations as the primary cause. Instead, translational efficiency affecting protein levels, cell membrane functions, cellular drug uptake, or efflux in general may contribute to the tolerance phenotype at higher 5FC concentrations (Fig. 5A). Therefore, gene dosage and/or resulting ectopic protein levels of the *FCY2.2 (B9J08_003017*) homolog may be another cause of 5FC resistance that warrants a future in-depth investigation.

SNP calling from transcriptomic data does not provide whole-genome coverage; however, it focuses on genes actively expressed under specific conditions (41). Currently, high-quality RNA-seq data of fungal research are often performed in triplicate or quadruplicate. The analyses of pooled data sets offer high read depth, enabling reliable genetic variant calling. While technical challenges such as alignment errors and limited genome coverage remain, variant calling from RNA-seq data offers the advantage of providing simultaneous insights into both gene expression and DNA variants (42). Furthermore, actively transcribed variants become apparent, potentially highlighting their functional relevance, an aspect that DNA-based approaches might overlook (42). Here, we validate three variants of *FUR1* in eight adapted clones using Sanger sequencing, confirming the results from RNA-seq. This highlights the reliability and usability of our workflow for variant calling from RNA-seq data (Fig. 2).

Although we demonstrate through a genetic reversal experiment that Q30* in *FUR1* promotes 5FC resistance in *C. auris*, certain limitations may still apply. For example, we did not systematically reverse all mutations identified through RNA-seq and WGS, such as the I212_A221delinsMAIRQMMAMK in *FCY2* or the ∆4nt175fs and R214T in *FUR1*. Future studies should focus on a comprehensive characterization of different *FUR1* and *FCY2* variants to map mutational landscapes that establish 5FC resistance in *C. auris*.

Taken together, the rapid emergence of resistant clones appearing upon 5FC treatment confirms that 5FC monotherapy remains problematic for treating *C. auris* infections (15, 20). Therefore, combination therapy is essential when using 5FC in clinical settings (14–16), but it requires efficient predictive tools to assess the likelihood of emerging clinical 5FC resistance. The serial sequencing of *C. auris* patient isolates, and modifications of the bioinformatic workflow developed here, can facilitate resistance monitoring to recognize and avoid clinical 5FC resistance. Notably, this bioinformatic workflow facilitates tracking of both transcriptional expression and genetic plasticity of clinical isolates from only RNA-seq data sets.

## MATERIALS AND METHODS

### Culture conditions and antifungal susceptibility testing

All *C. auris* strains were cultured on YPD (all from Formedium) agar and then inoculated into 2 mL of fresh prewarmed YPD liquid medium for 18 hours at 30°C with 200 rpm agitation before experiments. Roswell Park Memorial Institute medium (RPMI; Gibco, 21875091), supplemented with 0.165 M 3-(N-morpholino)propanesulfonic acid (MOPS; AppliChem, A2947) and adjusted to pH 7 (referred to as RPMI), was used for antifungal susceptibility testing assays (MIC assay). YPD medium was used for most experiments except the MIC assays.

Antifungal susceptibility testing was conducted adhering to CLSI guidelines (27). Briefly, fungal cells from overnight cultures were reinoculated into fresh YPD medium at an initial OD_600_ of 0.1 and incubated for 4 hours. 5FC (abcr GmbH, AB103900) was prepared as a twofold serial dilution in RPMI medium, ranging from 0.125 to 64 µg/mL. Fungal suspensions were prepared in RPMI medium and added to each well to reach a final density of approximately 2.5 × 10^3^ cells per well. The plates were then incubated at 37°C for 24 hours, followed by OD_600_ measurement with a Victor Nivo plate reader (PerkinElmer). A blank control well without fungal cells was included. MIC refers to the concentration of a drug that inhibits 50% growth when compared to the no-drug control.

### RNA sequencing analysis

Fungal cells from overnight cultures were diluted in fresh YPD medium to reach an OD_600_ of 0.1. The cells were then cultured at 37°C with agitation at 200 rpm until reaching an OD_600_ of 2.5. After incubation, fungal cells were collected by centrifugation at 3,000 × *g* for 3 minutes, resuspended in TRIZOL (LabConsulting, TS120) for RNA extraction, and disrupted using 200 mg glass beads (Sigma-Aldrich, G8772) with bead beating (FastPrep-MPI) at 6 m/s for 45 seconds, repeated twice.

Total RNA was isolated from cell pellets with chloroform, followed by precipitation with isopropanol at −20°C for 30 minutes. The RNA pellets were washed with cold 70% ethanol, air-dried, and subjected to DNase treatment with RNase-free DNase I (10U, Thermo Scientific, 10649890). RNA quality and integrity were assessed using a Bioanalyzer RNA 6000 Nanochip (Agilent Technologies), followed by mRNA enrichment with poly-T oligo-attached magnetic beads. Double-stranded cDNA libraries were prepared, pooled, and sequenced with 150 bp paired-end reads on the Illumina NovaSeq 6000 platform. RNA-seq data analysis was conducted using exactly the previously established workflow (https://github.com/kakulab/CSP2024) (15, 16). Gene Ontology (GO) enrichment analysis for cellular components (GO-CC) was performed by the enrichGO function and visualized as bar plots using the clusterProfiler R package (43), using annotation data of *C. albicans* retrieved from FungiDB v.54 (44).

### SNP variant calling from RNA-seq data

The bioinformatics pipeline was developed based on parts of *MycoSNP* (39, 45) available at github.com/kakulab/5FC-Evo-2024. Briefly, quality control of raw reads was carried out using FastQC v.0.11.9 (46). Adapter and low-quality sequences were trimmed from raw reads using TrimGalore v.0.6.6 (47). Trimmed reads were aligned to the genome assembly GCA_002759435.2 of B8441 strain (45, 48) as reference genome utilizing STAR’s two-pass mode v.2.6.1d (30) to enhance alignment accuracy, especially around novel splice junctions. BAM file filtering and indexing were performed using SAMtools v.1.9 (49). RNA-seq library quality control was implemented using RSeQC v.4.0.0 (50). Duplicate reads were marked using Picard tools v.2.23.9 (51). Library complexity was estimated using Preseq v.2.0.3 (52). Duplication rate quality control was performed using dupRadar v.1.18.0 (53). Reads overlapping with exons were assigned to genes using featureCounts v.2.0.1 (54). Classification of rRNA genes/exons and their reads was based on annotations and RepeatMasker rRNA tracks from UCSC Genome Browser when applicable. Genome coverage was calculated using Mosdepth v.0.3.8 (55). Following duplicate marking, the BAM files were recalibrated using truth SNP sets and a consensus variant call format (VCF) derived from whole-genome alignment with strain B8441 (48).

We implemented a bulk RNA data processing workflow for short variant discovery utilizing GATK v.4 (56) and associated tools (Fig. 2A). First, raw variants were called from post-processed BAM files using GATK HaplotypeCaller in genomic variant call format (GVCF) mode, followed by joint genotyping on all five strains. To minimize false-positive variants in the joint calling VCF, we applied hard filtering thresholds: QD < 2.0, MQ < 40.0, FS > 60.0, SOR > 3.0, MQRankSum < −12.5, and ReadPosRankSum < −8.0 (57). Any variant matching at least one of these criteria with an overall read depth of less than 30 was removed. To generate a highly confident annotation call set, we performed genotype masking on variants with a genotype quality below 50 and where less than 80% of reads mapped to the alternate allele in the corresponding sample. Genotypes of qualified variants remained unchanged. Additionally, functional annotation on the joint SNP calling was performed using SnpEff v5.2 (58). The sequencing depth of variants per sample was determined using the read depth information (FORMAT/DP field) from the annotated VCF file for the alternate allele. Furthermore, we evaluated genotype missingness by calculating the percentage of unmasked genotype calls that were missing or unidentified, both on a per-sample and per-variant basis. PCA was conducted using a linkage disequilibrium (LD)-based pruning method (31), which involved removing highly correlated SNPs, resulting in a representative subset that retains the majority of the genetic information. A pairwise distance matrix was computed using Pearson correlation coefficients with the pheatmap package (59). The selection of SNPs of interest focused on identifying variants that differentiate the resistant strains (R1, R2, and T1) from the susceptible strains (WT and S1).

### Genomic DNA extraction, PCR, and Sanger sequencing

Genomic DNA was extracted using the Monarch Genomic DNA Purification Kit (NEB, T3010) following the manufacturer’s protocol. The *FUR1, FCY2*, and *B9J08_004105* loci were amplified with primers indicated in Table S5. The PCR products were purified using the GeneJET PCR Purification Kit (ThermoScientific, K0702) and then sequenced using the Mix2Seq Kit NightXpress from Eurofins Genomics.

### Whole-genome sequencing

Genomic DNA was processed for quality control using Qubit 3.0 fluorometer and agarose gel electrophoresis. Library construction was performed using the VAHTS Universal Plus DNA Library Prep Kit for Illumina (ND617). Library fragment size and quality were assessed using the Qsep-400 capillary electrophoresis system. The prepared library was sequenced on the Illumina NovaSeq platform in PE150 mode at Biomarker Technologies (BMK) GmbH, Germany.

WGS bioinformatic analysis was conducted using the MycoSNPs pipeline published by the CDC (39). Variant filtration criteria include “QD < 2.0 || FS > 60.0 || MQ < 40.0 || DP < 10.” Additionally, genotype masking was performed to minimize false-positives or low-confidence variants by applying the following thresholds: a genotype quality is greater than or equal to 50, a minimum total read depth (DP) of 10, and minimum alternate allele fractions of 40% and 80%, respectively, in successive filtering. CNV analysis was also carried out using CNVkit (60). Subsequently, the parental WT strain was used to construct the copy number reference profile. Bin-level log_2_ ratios and segmented log_2_ ratios were calculated to identify copy number aberrations across scaffold regions, with an average bin size of 550 nucleotides.

### Multiple sequence alignments (MSAs) and protein modeling

All protein sequences were retrieved from UniprotKB. MSA was generated with the ClustalOmega method using the msa package in R (61). Phylogenetic trees were constructed based on these alignments using the “ape” and “ggtree” packages in R with the neighbor joining algorithm (62, 63). MSA results were visualized with ClustalX 2.1.

Homology models of *C. auris* Fur1 were established based on the template 1JLS (PDB) from *Toxoplasma gondii* (64) using the Swiss-Model tool (65). Models were visualized with UCSF ChimeraX version 1.8 (66).

### Genetic reversal for *FUR1*

A 1,312 bp fragment, including 627 bp upstream of *FUR1* and its ORF, was amplified from the genomic DNA of the WT strain. An additional 489 bp upstream region adjacent to *FUR1* was also amplified. The *NAT1* marker, including its promoter and terminator, was amplified from the plasmid pTS50 (67). These three fragments were purified using the GeneJET PCR Purification Kit and then connected through a Fusion PCR using Phusion High-Fidelity DNA Polymerase (ThermoScientific, F530), based on a 20 bp overlapping region added to the primers. The constructed DNA cassette, *FUR1*-upstream*FUR1*-pTEP-*NAT1*-tTEP-upstream*FUR1*, was then transformed into the strains R2 following the previously described protocol (29). Corrected clones were identified by colony PCR. Sequence of *FUR1* was confirmed by Sanger sequencing.

## Data Availability

RNA-seq data generated from this study were deposited via GEO database with accession number GSE272878. WGS data were deposited via the NCBI Sequence Read Archive (SRA) BioProject PRJNA1203645. The entire bioinformatic workflow was deposited via Github at https://github.com/kakulab/5FC-Evo-2024.

## SUPPLEMENTAL MATERIAL

Supplemental material msphere.00977-24-s0001.docx Figures S1 and S2; Tables S1 to S3; captions for Tables S4 to S7.

Supplemental tables msphere.00977-24-s0002.xlsx Tables S4 to S7.

ASM does not own the copyrights to Supplemental Material that may be linked to, or accessed through, an article. The authors have granted ASM a non-exclusive, world-wide license to publish the Supplemental Material files. Please contact the corresponding author directly for reuse.
