# Supplementary material for: Rapid in vitro evolution of flucytosine resistance in Candida auris
Source: mSphere. 2025 Mar 18;10(4):e00977-24. doi: 10.1128/msphere.00977-24 (PMC12039228; doi:10.1128/msphere.00977-24)
Supplement: Supplemental material — Figures S1 and S2; Tables S1 to S3; captions for Tables S4 to S7. [file msphere.00977-24-s0001.docx]

**Supplemental materials**


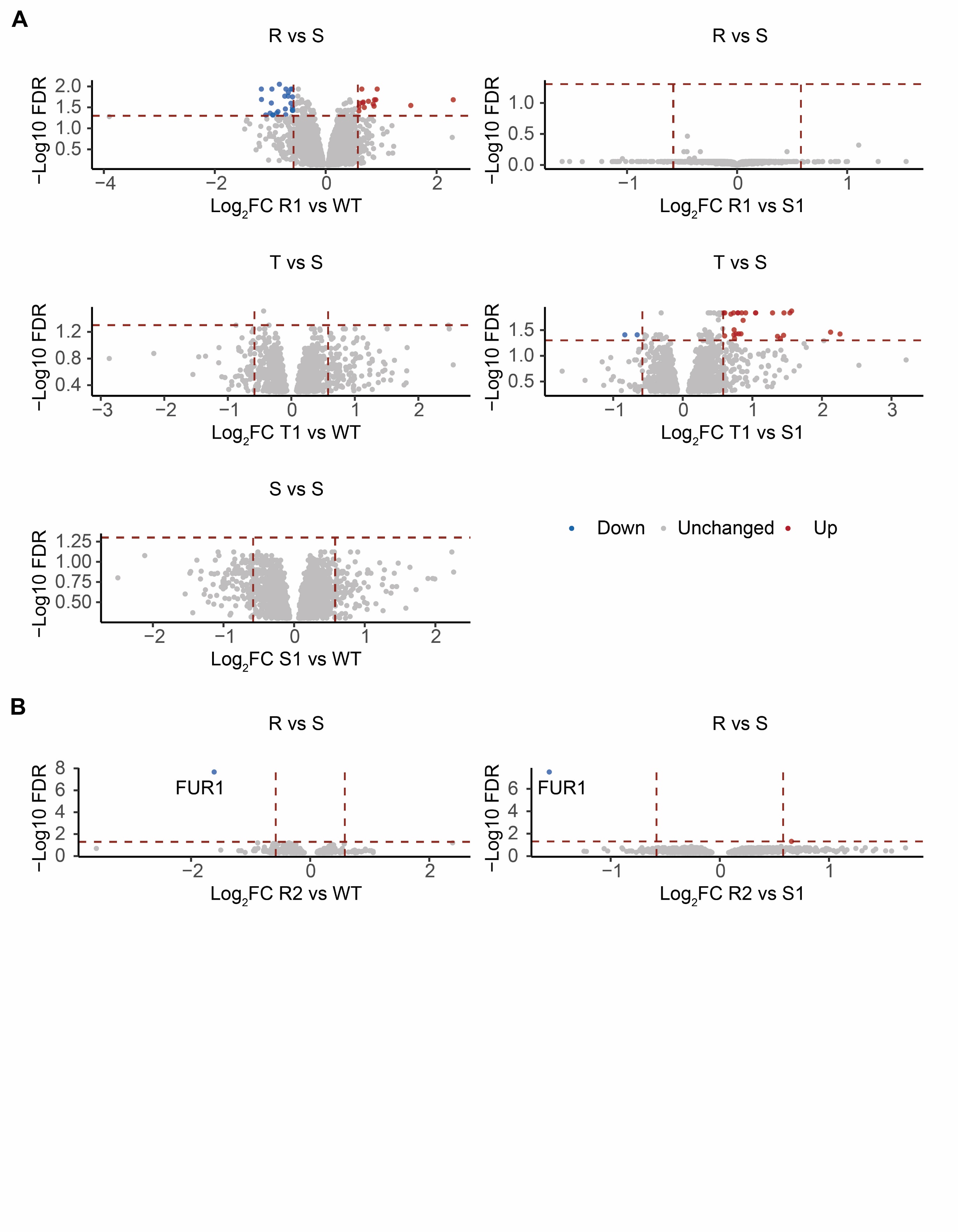


**Figure S1.** **Comparative transcriptome profiles of 5FC-resistant and sensitive phenotypes of *C. auris*.** R: resistant; T: tolerance; S: sensitive. Differential expression analysis performed using the EdgeR package.


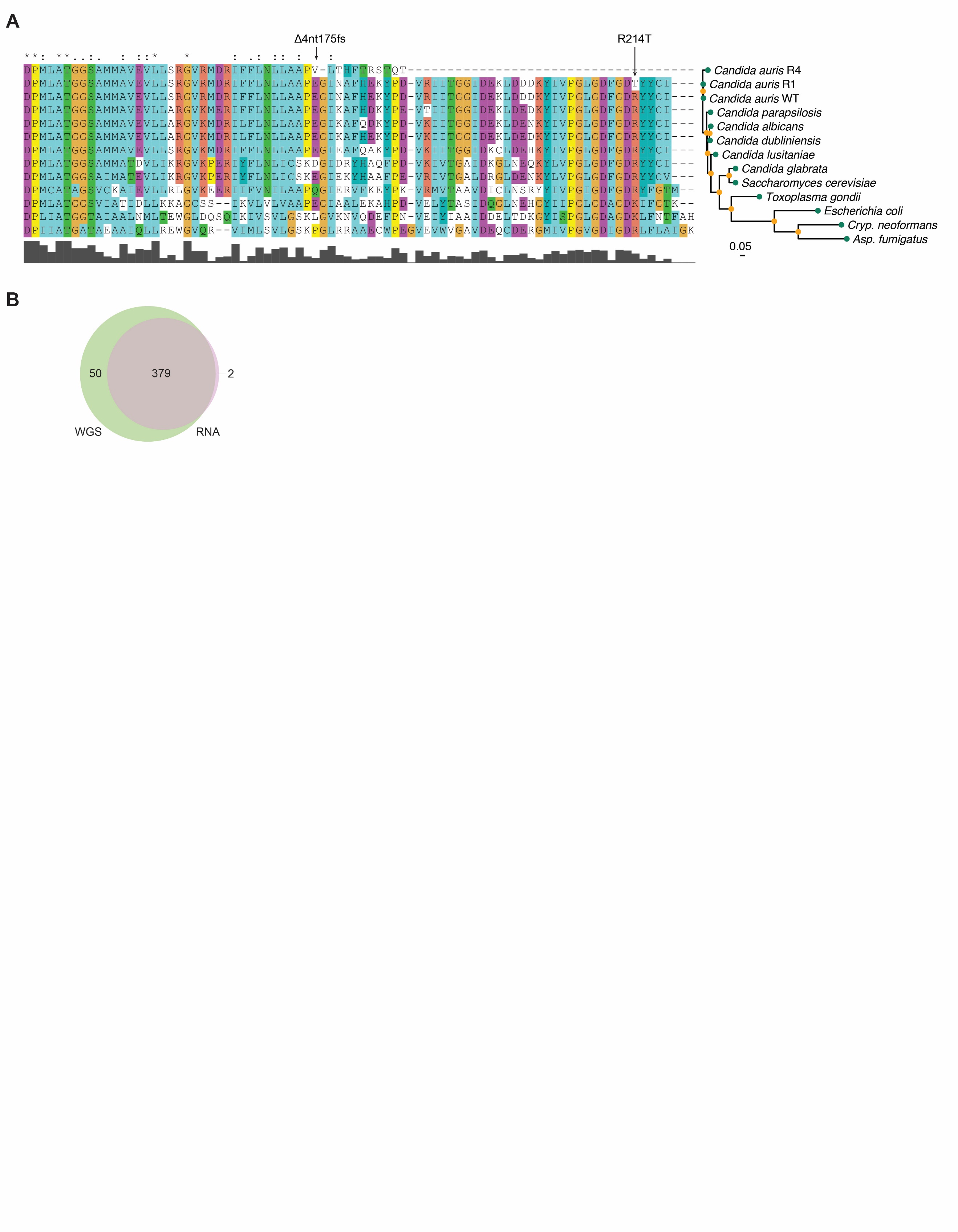


**Figure S2. A,** Multiple sequence alignment and phylogenetic tree of Fur1 across bacteria and fungi. Only the C-terminal Fur1 residues were visualized. **B,** The Venn diagram shows overlapping residue-altering mutations of the parental WT compared to the B8441 strain based on WGS and RNA-seq data, highlighting that most variants between both datasets are consistent.

**Table S1. Details of RNA-seq data alignments for *C. auris* clones after merging three replicates**

| **Strain** | **Phenotype** | **# reads (Millions)** | **# reads mapped** | **% Duplication** | **% rRNA** | **% Assigned** |
| --- | --- | --- | --- | --- | --- | --- |
| R1 | Resistance | 86.5 | 96.00% | 59.40% | 0.00% | 93.90% |
| R2 | Resistance | 93.4 | 95.90% | 59.60% | 0.00% | 93.60% |
| T1 | Tolerance | 94.1 | 95.90% | 58.50% | 0.00% | 93.80% |
| S1 | Susceptible | 82.5 | 96.00% | 58.20% | 0.00% | 94% |
| WT | Susceptible | 75.8 | 94.60% | 76.50% | 0.00% | 93.20% |

**Table S2. Read-depth summary of aligned reads**

| **Strain** | **Fraction of genome with at least 30X coverage** | **Median coverage** | **Mean coverage** |
| --- | --- | --- | --- |
| R1 | 88.60% | 375X | 820.6X |
| R2 | 89.50% | 411X | 880.9X |
| T1 | 90.00% | 430X | 911.4X |
| S1 | 88.60% | 372X | 805.5X |
| WT | 82.10% | 168X | 410.7X |

**Table S3. 5-FC corresponding mutation genes.** There are no differences between resistance and tolerance phenotype compared to WT in 5FC resistant associated genes.

| **Strain** | **Phenotype** | **Ref.** | **Mutations with residue changes in key target genes** | | | | | | **Data** |
| --- | --- | --- | --- | --- | --- | --- | --- | --- | --- |
|  |  |  | ***ADE17***  ***B9J08_001850*** | ***FCY1***  ***B9J08_005397*** | ***FCY2***  ***B9J08_002435*** | ***FCY2.2***  ***B9J08_003017*** | ***FCY23***  **B9J08_001737** | ***FCY24***  **B9J08_004513** |  |
| R1 | Resistance | B8441 | None | Ser70Arg | None | None | None | None | WGS RNA |
| R2 | Resistance | B8441 | None | Ser70Arg | None | None | None | None |  |
| T1 | Tolerance | B8441 | None | Ser70Arg | None | None | None | None |  |
| S1 | Susceptible | B8441 | None | Ser70Arg | None | None | None | None |  |
| WT | Susceptible | B8441 | None | Ser70Arg | None | None | None | None |  |

**Table S4.** Fungal strains used in this study*

**Table S5.** Primers were used in this study*

**Table S6.** Differential gene expression analysis from RNA-Seq data*

**Table S7.** Protein-altering mutations of the WT strain using B8441 as reference genome from RNA-seq and WGS datasets*

* These tables are included in the accompanying Excel file.
